# Supplementary material for: Content-rich biological network constructed by mining PubMed abstracts
Source: BMC Bioinformatics. 2004 Oct 8;5:147. doi: 10.1186/1471-2105-5-147 (PMC528731; doi:10.1186/1471-2105-5-147)
Supplement: Additional File 5 — The original Chilibot query results of the term "long-term potentiation (LTP)" and 22 other terms, limiting the latest references analyzed to the years 1990, 1995, 2000, and 2004. [file 1471-2105-5-147-S5.bz2 › chilibotAdditionalFile5/ltp2004/html/LTP_AMPA.html]

 


 **LTP** and **AMPA** 
  
Found 325 abstracts in PubMed,  **30 abstracts were retrieved and analyzed**.  


---

 Search Google  |
 PDF files only 
|  EDU domain only 

---

**Interactive relationship** (e.g. stimulation, inhibition, etc)

- An important question presents itself How do DA receptors influence glutamate dependent synaptic plasticity ? Alterations in  **AMPA**  receptor phosphorylation and trafficking are critical for  **LTP** .  Ref: 14684450 Ann N Y Acad Sci, 2003
- As judged by unaltered paired pulse facilitation, this  **LTP**  form is postsynaptically expressed despite depleted extrasynaptic  **AMPA**  receptor pools with reduced levels of GluR B immunoreactivity, which accumulates in somata and synapses of CA1 pyramidal neurons in GluR A deficient mice.  Ref: 14555717 J PhysiolJ Physiol, 2003
- In fact, glutamate receptor 1 GluR1 or GluRA subunits containing L alpha amino 3 hydroxy 5 methylisoxazole 4 propionate  **AMPA**  receptors have been shown to be required for several forms of hippocampal  **LTP**  and a particular hippocampal dependent learning task.  Ref: 12878757 J Physiol, 2003
- Moreover, long term potentiation  [ **LTP** ]  is associated with the formation of dendritic spines as well as synaptic delivery of  **AMPA**  receptors.  Ref: 12904794 Nature, 2003
- It is now well accepted that the trafficking of  **AMPA**  receptors to and away from the synaptic plasma membrane plays an essential role in both  **LTP**  and LTD, respectively.  Ref: 14684431 Ann N Y Acad Sci, 2003
- Together the data suggest that the highly regulated movement of GluR1 containing  **AMPA**  receptors between extrasynaptic and synaptic receptor pools is critically involved in establishing two functionally diverse forms of synaptic plasticity  **LTP**  and distance dependent scaling.  Ref: 12878757 J Physiol, 2003
- Dynamic movements of  **AMPA**  receptors in and out of the postsynaptic membrane account for, at least in part, the expression of NMDA receptor dependent changes in synaptic efficacy such as long term potentiation  [ **LTP** ]  and long term depression.  Ref: 12767475 Neurosci Res, 2003
- Changes in alpha amino 3 hydroxy 5 methyl isoxazole propionate  **AMPA**  receptor expression, localisation and electrophysiological responses should be induced by mechanical strain and inhibited by modulators of neuronal long term potentiation  [ **LTP** ] .  Ref: 12892570 BMC Cell BiolBMC Cell Biol, 2003
- Prior studies showed that positive alpha amino 3 hydroxy 5 methyl 4 isoxazolepropionic acid  **AMPA**  receptor modulators facilitate long term potentiation  [ **LTP** ]   **LTP**  and improve the formation of several types of memory in animals and humans.  Ref: 14751292 Neuroscience, 2004
- In adult mice, long term potentiation  [ **LTP** ]   **LTP**  of synaptic transmission at CA3 to CA1 synapses induced by tetanic stimulation requires L a amino 3 hydroxy 5 methyl 4 isoxazolepropionate  **AMPA**  receptors containing GluR A subunits.  Ref: 14555717 J PhysiolJ Physiol, 2004

**Parallel relationship** (e.g. studied together, co-existance, homology, etc.)

- Tunicamycin, thus, appears to serve as a modulator for NMDA and  **AMPA**  receptors, regardless of N glycosylation, thereby inhibiting neurotransmission and  **LTP**  in the dentate gyrus.  Ref: 12834891 Brain Res, 2003
- Activation of the enzyme phospholipase PLA 2 has been proposed to be part of the molecular mechanism involved in the alteration of 2 amino 3 hydroxy 5 methyl 4 isoxazole propionate  **AMPA**  glutamate receptor responsiveness during long term changes in synaptic plasticity long term potentiation  [ **LTP** ] .  Ref: 14561756 J Biol Chem, 2004
- Postsynaptic density 95 controls  **AMPA**  receptor incorporation during long term potentiation  [ **LTP** ]  and experience driven synaptic plasticity.  Ref: 14749436 J Neurosci, 2004
- These results suggest that 1 modulation of  **AMPA**  receptors which increases either response amplitude or duration can facilitate  **LTP**  formation, 2 modulators that effectively slow response deactivation augment the maximum magnitude of  **LTP**  and LTD, and 3 receptor desensitization may have a minor impact on synaptic plasticity in the hippocampus.Taken together, our data indicate that  **AMPA**  receptor modulators differ substantially in their ability to enhance synaptic potentiation or depression, depending on their particular influence on receptor kinetics, and hence that they may also be differentially effective in influencing higher order processes such as memory encoding.  Ref: 14751292 Neuroscience, 2004
- A juvenile form of postsynaptic hippocampal  **LTP**  in mice deficient for the  **AMPA**  receptor subunit GluR A.  Ref: 14555717 J PhysiolJ Physiol, 2004
- Activation of PI3 kinase is required for  **AMPA**  receptor insertion during  **LTP**  of mEPSCs in cultured hippocampal neurons.  Ref: 12765612 Neuron, 2003
- However, these mutant mice are competent in establishing several forms of long lasting synaptic changes in the CA1 region of the hippocampus, including LTD, long term potentiation  [ **LTP** ]   **LTP** , depotentiation, and dedepression, indicating the presence of GluR2 3 independent mechanisms of LTD expression and suggesting that  **AMPA**  receptor GluR1 alone is capable of various forms of synaptic plasticity.  Ref: 12848940 Neuron, 2003
- Spontaneous synaptic activity driven GluR B long transport maintains one third of the steady state  **AMPA**  receptor mediated responses, while GluR B long delivery following the induction of  **LTP**  is responsible for approximately 50% of the resulting potentiation at the hippocampal CA3 to CA1 synapses at the time of GluR B long peak expression the second postnatal week.  Ref: 14687553 Neuron, 2003
